# Public Health Literacy, Knowledge, and Awareness Regarding Antibiotic Use and Antimicrobial Resistance during the COVID-19 Pandemic: A Cross-Sectional Study

**DOI:** 10.3390/antibiotics10091107

**Published:** 2021-09-13

**Authors:** Suhaib M. Muflih, Sayer Al-Azzam, Reema A. Karasneh, Barbara R. Conway, Mamoon A. Aldeyab

**Affiliations:** 1 Department of Clinical Pharmacy, Faculty of Pharmacy, Jordan University of Science and Technology, Irbid 22110, Jordan; salazzam@just.edu.jo; 2 Department of Basic Medical Sciences, Faculty of Medicine, Yarmouk University, Irbid 21163, Jordan; reema.karasneh@yu.edu.jo; 3 Department of Pharmacy, School of Applied Sciences, University of Huddersfield, Huddersfield HD1 3DH, UK; b.r.conway@hud.ac.uk; 4 Institute of Skin Integrity and Infection Prevention, University of Huddersfield, Huddersfield HD1 3DH, UK

**Keywords:** public knowledge, attitudes, antibiotic resistance, health literacy, awareness, COVID-19

## Abstract

Multi-drug-resistant (MDR) organisms pose a global threat to modern medicine, which has grown as a result of irrational antibiotic use and misuse. This study aimed to assess general public knowledge in Jordan and awareness of antibiotics and antibiotic resistance during the COVID-19 pandemic. A cross-sectional study was carried out utilizing the WHO multicountry public awareness survey. The study population was composed mainly of social media users, and a total of 1213 participants completed the online survey. According to the findings, more than half of the participants were well versed in antibiotic use and resistance. Those with adequate health literacy were found to better understand antibiotics (OR = 1.37, *p* = 0.017) and antibiotic resistance (OR = 1.46, *p* = 0.003). The vast majority (88.5%) recognized at least one antibiotic resistance term; however, 53.2% believed that antibiotic resistance is a problem in other nations. The participants in this study reported using antibiotics incorrectly, believing that they were treating sore throats, colds, and flu. The participants were well aware of antibiotic resistance solutions and their consequences on health. Age, education, health literacy, and antibiotic knowledge were found to be substantially (*p* < 0.05) associated with greater awareness of antibiotic resistance. The findings highlight the need for antimicrobial resistance education campaigns, health literacy, and antibiotic stewardship initiatives.

## 1. Introduction

The emergence of multi-drug-resistant (MDR) organisms has posed a global challenge to modern medicine since the discovery of antibiotics. Antibiotic resistance occurs naturally, but unsustainable antibiotic use and misuse have expedited the process, putting humanity’s well-being in danger [1,2,3]. An estimated 700,000 people die annually as a result of antimicrobial resistance (AMR), which is defined as bacteria surviving under the influence of antimicrobial agents [4,5]. Healthcare systems and the global economy have been challenged since COVID-19 was declared a global pandemic. For the clinical management of COVID-19, increased use of empirical antibiotics in patients with respiratory symptoms has been reported [6,7,8]. These practices have the potential to amplify AMR and MDR pathogens [9,10,11,12]. Studies have associated higher education with a better understanding of antibiotic resistance [13,14]. Antibiotic resistance awareness can be considerably increased by raising people’s knowledge and health literacy, which will have a positive impact on actions such as irrational antibiotic dispensing and self-medication [15,16]. Health literacy is defined by the World Health Organization (WHO) as the ability to make effective health decisions in order to increase human health [17]. According to prior research, people who live in cities have higher levels of health literacy than those who live in rural areas [18,19]. Similarly, an Egyptian cross-sectional investigation linked antibiotic resistance awareness to health literacy. According to the study, 92% of people were unaware of antibiotic resistance, and 40% had used antibiotics without a prescription in the previous month [20]. Community pharmacies are the most common form of public primary care in Jordan, providing prescription and nonprescription medications for a wide range of conditions. Despite Jordanian regulations prohibiting the dispensing of antibiotics without a prescription, studies found that more than 40% of participants purchased antibiotics from community pharmacies without a prescription [21,22,23,24]. The majority of participants who self-medicate obtained antibiotics and sought medical advice mostly from pharmacies before contacting prescribing physicians. Previous research found that more than half of Jordanians improperly use antibiotics to treat common colds and coughs, sore throats, and influenza [21,22,24]. As a result, the lack of awareness and the broad availability of antibiotics lead to an increase in inappropriate self-medication and, as a result, antibiotic misuse among Jordanians. In a cross-sectional study conducted in an outpatient hospital environment in Jordan, Muflih et al. (2020) reported that 74.2% of patients self-medicate with antibiotics. The study found a significant association between overall health literacy and inappropriate self-medication, highlighting the importance of raising awareness and improving health literacy [25]. According to studies, the majority of interviewees reported self-medicating with antibiotics and considered it a safe and acceptable practice [26,27,28]. In addition, the increased use of antibiotics in outpatient settings could be related to lack of awareness and external patient pressure [29]. Of note, a key driver to rising antimicrobial resistance, according to Laxminarayan et al. (2013) and Pereko et al. (2015) [30,31], is the misuse of antibiotics for self-limiting diseases, such as cold and influenza, sore throat, and diarrhea. Both incorrect dosing and the use of antibiotics when they are not required significantly contribute to the rising problem of antibiotic resistance [32,33]. Despite the fact that the world’s attention is currently focused on the global COVID-19 epidemic, the AMR issue should also be prioritized [34], and research studies examining public perceptions of AMR during the present pandemic are needed. Therefore, this process was undertaken in Jordan during the COVID-19 pandemic to assess the general public’s knowledge and awareness regarding antibiotics and antibiotic resistance.

## 2. Results

### 2.1. Characteristics of Study Participants

The study included 1213 participants, the majority of whom were females (*n* = 856; 70.6%). Almost one-third of the participants (35.4%) were between the ages of 25 and 34, and the vast majority lived in cities (61.1%). Around half of the participants (54.9%) were educated with a bachelor’s degree, and less than one-third (28.3%) were employed in the medical field. Additional sociodemographic factors are listed in Table 1.

### 2.2. Participants’ Health Literacy Levels

According to the Single Item Literacy Screener (SILS), when asked “how often do you ask someone for help reading instructions and leaflets from a doctor or pharmacy”, the participants said “1 = always” (*n* = 28, 2.3%), “2 = most of the time” (*n* = 94, 7.7%), “3 = sometimes” (*n* = 332, 27.4%), “4 = rarely” (*n* = 403, 33.2%), and “5 = never” (*n* = 356, 29.3%). The findings of this study indicated that nearly two-thirds (62.6%) of the participants possessed adequate health literacy, while 37.4% had inadequate health literacy. With the exception of education level, health literacy levels were comparable across groups. Consistent with previous research [15,31], this study revealed that people with a postgraduate degree were more likely than those with lower education levels to report better health literacy, which may represent the influence of education on participants’ exposure to and evaluation of health information essential to maintain their health.

### 2.3. Knowledge of Antibiotic Use among Participants

According to the findings, 81.5% correctly stated that patients should discontinue taking prescription antibiotics after taking them as prescribed. However, 17.1% believed that antibiotics should be discontinued after they felt better. Despite the fact that antibiotics can be used to treat similar conditions, 82.2% of the respondents felt that using antibiotics given to them by a friend or family member is unacceptable. Furthermore, 41% believed that it was appropriate to obtain or request the same medications from a doctor in order to treat the same symptoms they had previously encountered. When asked about particular infections that might be treated with antibiotics, the majority of the participants correctly identified only two conditions: bladder/urinary tract infections (75.4%) and skin/wound infections (66.4%). However, less than 14% properly classified gonorrhea and malaria as antibiotic-susceptible infections. According to 43.2% and 84.2% of the respondents, antibiotics can be used to treat colds/flu and sore throats, respectively (Table 2).

### 2.4. Knowledge of Antibiotic Resistance among Participants

The majority of the participants (85.8%) incorrectly identified the following statement as true: “Antibiotic resistance occurs when your body becomes resistant to antibiotics and they no longer work as well,” and over half (53.2%) wrongly believed antibiotic resistance is a concern in other nations but not in Jordan. Furthermore, 57.2% incorrectly believed that antibiotic resistance is limited to people who routinely take antibiotics, whereas only 37% believed that resistant bacteria can spread from person to person. Furthermore, more than half of those surveyed correctly identified that many infections are becoming increasingly resistant to antibiotic treatment, and those antibiotic-resistant infectious agents could make medical procedures far more dangerous. The majority of the participants were familiar with the following terms: “antibiotic resistance”, “superbugs”, “drug resistance”, and “antibiotic-resistant bacteria”, while 11.5% of those surveyed had never heard of any of these terms (Table 3).

### 2.5. Pattern of Antibiotic Usage in Relation to Knowledge of Antibiotics and Antibiotic Resistance

The survey results identified a correlation between individuals’ antibiotic consumption patterns and their overall knowledge of antibiotics and antibiotic resistance. Respondents who reported using antibiotics in the previous 6 months or longer had a higher likelihood of having adequate antibiotic knowledge (adjusted OR = 1.74, 95% CI: 1.294–2.35) and adequate antibiotic resistance knowledge (adjusted OR = 1.42, 95% CI: 1.06–1.91) than those who used antibiotics in the previous month; estimates for greater than 6 months are presented in Table 4. Further, respondents who reported receiving antibiotics through a healthcare provider’s prescription were more likely to have adequate knowledge of antibiotics (adjusted OR = 2.01, 95% CI: 1.57–2.58) and adequate knowledge of antibiotic resistance (adjusted OR = 1.31, 95% CI: 1.03–1.68) than those who self-medicate. Respondents who received healthcare provider counseling and instructions were more likely to have adequate antibiotic knowledge (adjusted OR = 1.98, 95% CI: 1.53–2.56) than those who did not (Table 4).

### 2.6. Factors Associated with Participants’ Knowledge of Antibiotics and Antibiotic Resistance

After identifying factors (such as age, gender, residence, family structure, education level, employment status, family monthly income, health insurance, and infection with COVID-19) associated with adequate antibiotic knowledge and antibiotic resistance using univariate logistic regression, variables with *p* < 0.25 were included in the multiple logistic regression model for further analysis. The findings showed that younger and female respondents and those who resided in cities were more likely to have adequate knowledge about antibiotics than their counterparts. Respondents working in the medical field (adjusted OR = 3.08, 95% CI: 2.13–4.44, and adjusted OR = 2.78, 95% CI: 2.00–3.87) were also more likely to have adequate knowledge of antibiotics and antibiotic resistance, respectively. Furthermore, participants with health insurance (adjusted OR = 1.38, 95% CI: 1.03–1.85) were more likely to be knowledgeable about antibiotic resistance than those with no health insurance (Table 5). Participants who did not get antibiotics (adjusted OR = 0.31, 95% CI: 0.22–0.45) to treat COVID-19 and those who received COVID-19 vaccination (adjusted OR = 1.62, 95% CI: 1.02–2.18) both showed a high degree of antibiotic knowledge. Participants with appropriate health literacy were more likely to have an adequate understanding of antibiotics (adjusted OR = 1.37, 95% CI: 1.06–1.77) and antibiotic resistance (adjusted OR = 1.46, 95% CI: 1.14–1.89) than their counterparts. Family structure, education level, and family monthly income were not correlated to an adequate degree of knowledge on antibiotics and antibiotic resistance (see Table 5).

### 2.7. Level of Awareness of Possible Solutions to the Problem of Antibiotic Resistance

**Figure 1:**
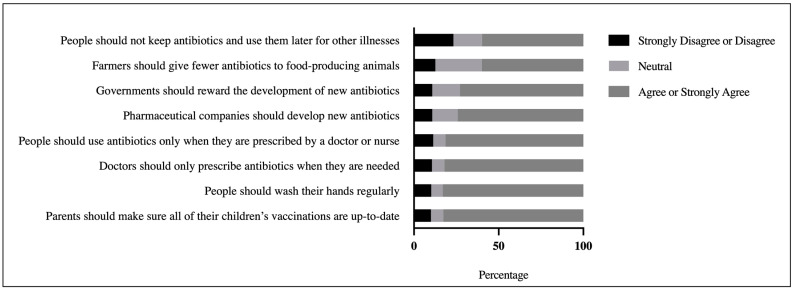
Participants’ awareness of possible solutions to the problem of antibiotic resistance.

The respondents were asked to rate their awareness of approaches to addressing the problem of antibiotic resistance. The findings revealed that the participants were well aware of the resistance issue (M = 3.91 out of 5, SD = 0.97). The majority of the respondents agreed or strongly agreed that practically all antibiotic-resistance-fighting measures, such as parents ensuring that all of their children’s vaccines are up-to-date (82.9%) and doctors giving antibiotics only when required (82%), were essential. However, the least agreed-upon advice was that farmers use fewer antibiotics in food-producing animals and that people should not stockpile antibiotics to use later for other diseases (Figure 1). The findings of the *t*-test demonstrated that levels of health literacy, antibiotic knowledge, and antibiotic resistance knowledge all had a significant effect on overall awareness of potential antibiotic resistance solutions. The results of one-way ANOVA also demonstrated that age and education level had a significant effect on total awareness score (Table 6). The findings of post hoc Tukey tests showed that individuals from the age groups 18 to 44 were much more aware of potential antibiotic resistance solutions than those aged 45 and older. The postgraduate education group participants were more aware than the other groups.

### 2.8. Participants’ Perspectives on the Scope of the Antibiotic Resistance Problem

**Figure 2:**
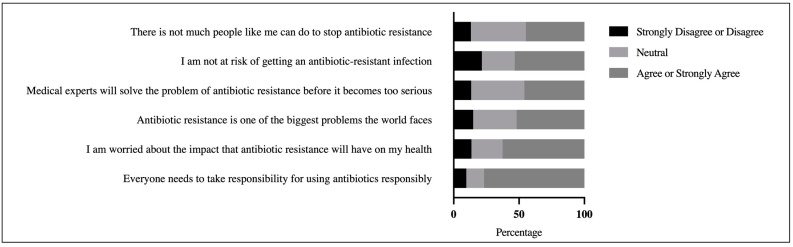
Participants’ perspectives on the scope of the antibiotic resistance problem.

According to the findings, the participants’ general perceptions of the magnitude of the antibiotic resistance problem were moderate (M = 3.51 out of 5, SD = 0.73). The majority (76.8%) agreed or strongly agreed that everyone must bear responsibility for safely taking antibiotics. In addition, 62.6% were concerned about the impact of antibiotic resistance on their own and their family’s health. Despite these beliefs, however, 53% of the respondents asserted that they are not in danger of acquiring an antibiotic-resistant infection if they adhere to their antibiotic regimen. There is substantial uncertainty over whether the issue of antibiotic resistance necessitates global collaboration. As illustrated in Figure 2, one-third or more of the respondents were undecided about whether antibiotic resistance is one of the world’s most serious concerns (33.2%), and 40.6% were undecided about whether medical specialists will address the problem of antibiotic resistance before it becomes too serious. The *t*-test results revealed that degrees of health literacy, antibiotic knowledge, and antibiotic resistance knowledge all had a significant effect on the overall perspective score (see Table 6).

## 3. Discussion

### 3.1. Health Literacy

The current study investigated the levels of health literacy in Jordanian communities in relation to their knowledge and awareness of antibiotics and antibiotic resistance. Almost two-thirds (62.6%) of those surveyed had adequate health literacy, which is slightly higher than the 57.2% previously reported in a cross-sectional study on the impact of health literacy on self-medication practice [25]. In line with earlier research [16,35], the findings of this study showed that health literacy levels were comparable across groups, with the exception of the education levels, which had a substantial effect on health literacy. This finding implies that higher education levels provide more broad exposure to antibiotic and health-behavior-related information. According to the findings of this study, 721 (59.4%) and 631 (52%) of the participants had adequate knowledge of antibiotic use and antibiotic resistance, respectively. Knowledge scores were significantly higher for those with adequate health literacy than those with inadequate health literacy (OR = 1.37 (1.05–1.77), *p* = 0.017; OR = 1.46 (1.14–1.89), *p* = 0.003), suggesting that overall health literacy is a key focus that crosses all disciplines.

### 3.2. Knowledge of Antibiotics and Antibiotic Resistance

According to the findings of this survey, more than half of the participants have adequate understanding of antibiotics and antibiotic resistance. Levels of knowledge differ significantly based on the participants’ age, gender, employment status, insurance, health literacy, and infection with COVID-19. The term “antibiotic resistance” has been heard by almost three-quarters of the participants, suggesting that antibiotic resistance is a frequently discussed issue that the vast majority of Jordanians are aware of. Similar findings were reported for all the participating countries in the WHO multicountry public awareness survey [2]. The participants in this study, however, frequently mischaracterized antibiotic resistance, such as “antibiotic resistance occurs when your body becomes resistant to antibiotics and they no longer work as well”, “antibiotic resistance is an issue in other countries but not in my country”, and “antibiotic resistance is only a problem for people who take antibiotics on a regular basis”. Acquainting patients with the fact that antibiotic resistance is attributed to bacteria’s capacity to overcome the action of therapeutic agents and that it is a global issue making everyone susceptible to antibiotic-resistant, difficult-to-treat infections may enhance social contributions to the implementation of antibiotic resistance prevention strategies. Previous studies reported similar results [2,20,36]. The findings of this study revealed that more than four-fifths (83%) of the participants made wrong assumptions regarding whether antibiotics heal sore throats, whereas less than half (43%) were incorrect in their assertion that antibiotics can be used to treat colds and the flu. This might be attributed to their lack of knowledge about the appropriate use of antibiotics as well as their lack of awareness regarding available alternatives that might help alleviate the symptoms of viral infections. Pereko et al. (2015) reported similar findings [31]. In contrast to the findings of Mostafa et al. (2021) [20], smaller percentages wrongly believed that antibiotics treat fever (24.6%) and diarrhea (16.7%).

The findings also revealed that more than two-thirds of the patients correctly believed that antibiotics are effective in treating urinary tract infections and skin infections; similar findings were observed by the WHO multicountry survey [2]. However, most participants were unaware that antibiotics can be used to treat gonorrhea and measles. These data suggest that the general population has conflicting understandings of antibiotic use. Solving these knowledge conflicts by promoting patient-centered interaction and developing antibiotic resistance education may help to combat antibiotic resistance and enhance public health by driving individuals to seek more accurate health-related information.

#### 3.2.1. Factors Associated with Participants’ Knowledge of Antibiotics and Antibiotic Resistance

According to the findings of this study, lack of knowledge of antibiotic use was more prevalent among older and male participants, as well as those who resided in rural areas and those who were unemployed or worked in nonmedical occupations. The findings suggest that older persons have poor comprehension of antibiotics. Similar findings were reported by other countries [37,38,39]. In contrast to the findings of Kong et al. (2019) [37], this study implies that men and women are disproportionately concerned about antibiotics. This could be because women are more likely to use primary healthcare services, are more experienced with antibiotic treatment, and take greater responsibility for their family’s health [40,41]. The findings mirror those of a recent study that found that rural residents possessed less knowledge about antibiotics than those who resided in cities [19]. This could be due to lack of access to healthcare and health insurance coverage. This study’s findings are in line with those of Haque et al. (2019), supporting the fact that working in medical disciplines is strongly associated with increased knowledge and learning capacities regarding antibiotic effectiveness and proper antibiotic use [26]. This study’s findings indicate that medical field participants have access to antibiotic courses at their institutions. While physicians should graduate from medical schools with the knowledge necessary to make sound decisions about antibiotic use, this issue should be given equal weight in nonmedical field studies all across Jordan. Comparisons among healthcare workers warrant further evaluations in future studies.

Further, the participants in this study who did not have health insurance had lack of knowledge about antibiotic resistance, which could imply that they are less likely to learn more about antibiotics and antibiotic resistance and are more likely to engage in less responsible self-medication as a result of economic restrictions. The incorrect and excessive use of broad-spectrum antibiotics in COVID-19 patients has been documented to outnumber the prevalence of bacterial coinfections and secondary illnesses [6,7,8]. However, this study’s findings indicate that individuals who did not get antibiotics to avoid the COVID-19 infection and those who received COVID-19 vaccine have a greater level of knowledge about antibiotics. This suggests that the respondents are aware of COVID-19-related information that was widely disseminated to the general population and have a higher degree of health literacy. Further, the participants with low health literacy were also shown to have lack of knowledge about antibiotics and antibiotic resistance, which may be a result of their inability to evaluate information received from healthcare practitioners. Similar findings were observed in Egypt, where low levels of antibiotic knowledge and awareness of antibiotic resistance were connected to low levels of health literacy [20].

#### 3.2.2. The Use of Antibiotics

The percentage of the respondents in this study who reported using antibiotics in the previous month, in the previous 6 months, and a year ago (28.4%, 30.1%, and 9.8%, respectively) was lower than that of the general public in low-income countries (e.g., Egypt, Sudan, and Indonesia), as reported by a WHO multicountry survey [2]. In comparison with prior studies conducted in Jordan [25,42], the current study’s finding of a general decrease in antibiotic use can be attributed to the increased level of knowledge of antibiotics and antibiotic resistance, stringent public health measures (e.g., promoting social distancing measures), and increasing antibiotic use and resistance awareness programs. Additionally, as a result of public health prevention initiatives and the availability of telemedicine, people are attending outpatient clinics less frequently [43]. Additional research is needed to determine the importance of antibiotic stewardship interventions, particularly those that involve remote monitoring, in order to optimize antibiotic use and infection treatment appropriateness.

Although a lower percentage of participants (30%) in this study reported inappropriate self-medication practices compared with a previous household study in Jordan (39.5%) [23], they demonstrated lack of understanding about antibiotic use and resistance compared with those who obtained antibiotics via a doctor’s or nurse’s prescription. These findings underscore the importance of conducting additional studies into the reasons for self-medication in order to increase public awareness and guide practice among the general public and healthcare professionals. Consistent with previous research, approximately half of those surveyed in this study indicated that pharmacies are their primary source of self-medicated antibiotics [13,26,44]. While pharmacists are responsible for promoting safe and cost-effective drug use, the study findings support previous research emphasizing the pharmacist’s role in referring patients with infectious diseases to physicians, advocating for prudent antibiotic use, and preventing antimicrobial resistance [13,35,45].

#### 3.2.3. Implications for Education Campaigns

In light of the current study’s findings and those of previous studies [4,30,46,47], ongoing educational activities should emphasize the importance of improving health literacy and information-seeking behavior to enhance health outcomes and to educate participants about the proper use of antibiotics, which may help curb the spread of antibiotic resistance. To increase public awareness of antibiotic resistance, efforts and educational initiatives should be directed toward the elderly, uninsured, those who self-medicate with antibiotics, and those with low health literacy. Additionally, a community-based health education program targeting various sections of the community would be the most effective strategy to educate people about judicious antibiotic usage.

### 3.3. Participants’ Awareness of Possible Solutions to the Problem of Antibiotic Resistance

Previous research revealed that the Jordanian community has a general lack of information and awareness about antibiotic use and antibiotic resistance [42]. The current study, on the other hand, found that the participants were well aware of potential solutions to the problem of antibiotic resistance. These disparities may be explained by a high level of health literacy, as well as the effectiveness of regular media messaging against antibiotic use during COVID-19, which may have served to expand knowledge. The participants in this study agreed or strongly agreed that physicians should prescribe antibiotics only when they are deemed necessary, and people should use antibiotics only when they are prescribed by a doctor or nurse, which was also reported in previous studies from other countries regarding overuse, underuse, and inappropriate use of antibiotics [2,48]. The positive attitude among the participants suggests better knowledge of antibiotic use and resistance. However, there was some confusion regarding some items, such as “Farmers should give fewer antibiotics to food-producing animals” and “People should not keep antibiotics and use them later for other illnesses”, which were the least agreed upon by the participants to address the problem of antibiotic resistance. Previous research yielded similar results [2,20]. The findings of this study propose instant solutions and various techniques to improve antibiotic stewardship in various livestock sectors. While boosting understanding regarding antibiotic use and resistance may be beneficial in raising awareness, methods such as employing nonantibiotic treatments, such as herbs and probiotics, or vaccinating herds may have a positive impact on limiting antibiotic resistance [49,50]. In line with earlier findings [2,20], about two-thirds of the participants in this study believed that the problem of antibiotic resistance is more likely to be avoided if many investments were dedicated to antibiotic research and development by the government and pharmaceutical companies (see Figure 1). While governments and pharmaceutical companies should develop novel approaches and interventions to address antimicrobial resistance, increasing public engagement and awareness to promote rational drug use could be critical in combating antimicrobial resistance in humans and animals. Although incorrect dosing and the use of antibiotics when they are not needed both contribute to the rising problem of antibiotic resistance [32,33], nearly half of the participants expressed misconceptions, believing that antibiotic-resistant infections could be avoided by taking antibiotics as prescribed, which may reflect lack of understanding of antibiotic resistance.

The participants’ awareness of potential solutions to the problem of antibiotic resistance differed significantly across age groups, education levels, health literacy levels, levels of knowledge of antibiotics, and levels of knowledge of antibiotic resistance. The findings could be due to the fact that younger participants and those with greater levels of education had more information about antibiotic resistance than their counterparts [13,14]. In line with previous studies [15,16], this study concludes that raising people’s knowledge and health literacy can significantly improve their awareness of antibiotic resistance, which can have an impact on practices such as irrational dispensing and self-medication. The lack of connections between gender and levels of awareness could indicate that individuals of both genders have similar views about the problem of antibiotic resistance. Similarly, the lack of any connections between the region of residence, work status, and awareness levels may signal that people have received sufficient messages aimed at increasing awareness and are paying close attention to the problem of antibiotic resistance.

### 3.4. Participants’ Perspectives on the Scope of the Antibiotic Resistance Problem

When asked about their opinions on antibiotic resistance and how it might affect their health, almost 63% were worried about the impact of antibiotic resistance on their health and their family’s health; similar results were reported in previous studies [2,20]. These findings may reflect a higher level of antibiotic awareness among the general public, as well as individual responsibility in the fight against antibiotic resistance. However, a study conducted in Malaysia reported a much lower percentage (48.5%) for perceived impact of antibiotic resistance on the participants and their family. Contrary to findings reported by previous studies [2,20,51,52], an intriguing research finding in this study was that only 52% of the participants recognized antibiotic resistance as one of the greatest challenges the world faces today. The main reason could be attributed to lack of knowledge as only 46.8% of the participants believed that antibiotic resistance is an issue that is not limited to Jordan but also concerns other countries. Thus, raising public knowledge and awareness through social and mass media could help in recognizing antibiotic resistance and the perceived threat it poses [53]. In this study, the participants with higher levels of knowledge and health literacy had better perspectives on the breadth of the antibiotic resistance problem than those with lower levels of knowledge and health literacy. This finding could be attributable to the effect of health information on favorable health-related behaviors, which could have a positive impact on responsible antibiotic use. Furthermore, participants with higher health literacy may be more aware of the potential negative repercussions associated with incorrect antibiotic usage and inaccurate diagnosis as reported recently elsewhere [16,54].

### 3.5. Limitations and Strengths

A convenience sampling method was applied to select participants based on their availability and willingness to participate in the online survey. However, this study’s findings have a number of limitations. Recruiting participants through social media is susceptible to a nonresponse bias due to the digital divide-unequal access to the Internet and disparities in usage style and frequency of social media; thus, the characteristics of nonresponders might differ from those of responders [55,56]; however, the survey link has been repeatedly posted and tagged to friends on Facebook to maximize the response rate, thus minimizing the possibility of nonresponse bias. There was an over-representation of younger respondents with more females and more unemployment that did not perfectly represent the target population according to Jordan’s census administrative records [57]. Therefore, the findings of this study could fall short in representativeness. Moreover, participants who might be concerned with the antibiotic topic could be more likely to take the survey, which might lead to self-selection [58]. This study defined health literacy as the perceived ability to read health-related information, as measured by the Single Item Literacy Screener (SILS), which could not detect literacy difficulties in those with modest reading abilities, and it is subject to false negative replies [59]. Moreover, the impact of chronic diseases on escalating antibiotic consumption was not a focus of this study. The study’s strength is that it involved a large number of participants, which enables the assessment of the relationship between knowledge, awareness, and antibiotic use in relation to a variety of sociodemographic factors. Given the scarcity of studies in Jordan on this topic, these findings are particularly useful and fill a gap in the literature by establishing a baseline of Jordanians’ knowledge, perspective, and behavior toward antibiotics.

In conclusion, while this study provides preliminary evidence of public awareness of antibiotic resistance and common antibiotic-resistance-related behaviors in Jordan, planning a future study with a more representative sample would help in generalizing the collected data to the larger population and confirming the validity of the study findings. To counteract and alleviate the severity of antibiotic resistance, public knowledge and awareness about antibiotic use should be increased. The vast majority of people were aware of antibiotics and antibiotic resistance, as well as their potential consequences on health. In the Jordanian community, there were widespread misconceptions about appropriate antibiotic use and behavioral components of antibiotic resistance. Antibiotic awareness was found to be associated with a high level of education, adequate health literacy, and adequate knowledge of antibiotics and antibiotic resistance. Future multicenter, well-designed prospective research studies should investigate the impact of chronic illnesses and patient–physician interactions on knowledge and its implications for responsible antibiotic use. Other programs should address the issue of self-medication and antibiotic resistance by addressing the entire spectrum of health literacy.

## 4. Materials and Methods

### 4.1. Study Design and Setting

A cross-sectional study design was carried out among Jordanians utilizing a convenience sample approach. The general public was invited to participate in an online self-administered questionnaire via an electronic invitation disseminated on social media (e.g., mainly Facebook). The survey link was frequently shared on Facebook in order to enhance response rates. The following criteria were used to identify eligible participants: (a) is a Jordanian citizen, (b) possesses adequate Arabic language abilities, (c) is 18 years of age or older, and (d) is willing to participate voluntarily in this study. A sample size of 1100 individuals was estimated using Raosoft software to attain the needed precision at a 95% confidence level, a 50% response distribution, and a 3% margin of error [60]. Responses to the study survey were automatically collected in Google Forms. The survey links redirected Facebook users to the survey’s first page, which contained thorough instructions regarding the study’s goal and expected outcomes, prior to participants consenting to participate and gaining access to the survey items. It was mentioned unequivocally that participation was completely voluntary and that confidentiality and anonymity would be maintained throughout the study. For data organization and analysis, Google Forms was linked to a Google spreadsheet.

### 4.2. Survey Instrument

A 47-item online survey questionnaire was employed in this study. At the beginning of the questionnaire, nine sociodemographic characteristics (i.e., age, gender, residence, family structure, education level, employment status, family monthly income, health insurance, and infection with COVID-19) were included. Participants of both genders were recruited on the basis of their age (>18 years) and geographic location (urban, suburban, and rural). The participants were asked about their family structure, which was classified as a nuclear family composed of parents and their children; a joint family composed of sets of siblings, their wives, and their dependent children; or an extended family composed of at least three generations. Employment status was also classified into three categories to ascertain whether the participants were unemployed, defined as unable to secure a paid job or out of paid work; employed in a nonmedical field, defined as any occupation that does not require a degree in a health-related field; or employed in a medical field, defined as any occupation that requires a degree in a health-related field (e.g., medicine, pharmacy, public health, clinical laboratory sciences, or nursing). The respondents’ educational attainment was operationalized as having completed high school, a bachelor’s degree, or a postgraduate degree (e.g., MSc or Ph.D.). The family monthly income of the participants was defined as the sum of the monthly household incomes of all family members who live together, which was classified as follows: less than JOD 500 (USD 700), between JOD 501 (USD 700) and JOD 1000 (USD 1400), between JOD 1001 (USD 1400) and JOD 1500 (USD 2100), and greater than JOD 1500 (USD 2100). Additionally, the respondents were asked whether they had health insurance at the time of the survey. Finally, the participants were asked whether they had previously been infected with COVID-19. The Single Item Literacy Screener (SILS) (2006) developed by Morris et al. (2006) [61] was adapted to serve as a predictor of the participants’ overall health literacy. The participants were asked, “How frequently do you seek assistance in reading the instructions and leaflets from your doctor or pharmacy?” The replies to the preceding question were scored using a 5-point Likert scale (5 = never, 4 = rarely, 3 = sometimes, 2 = often, 1 = always).

The World Health Organization’s (WHO) validated antibiotic resistance: multicountry public awareness survey was adapted in this study [2]. The WHO multicountry public awareness survey has a total of 37 items that assess people’s knowledge about antibiotics and antibiotic resistance, as well as their awareness of alternative solutions to antibiotic resistance and its health impacts. Antibiotic knowledge scores assess the levels of understanding around the appropriate use of antibiotics, including how and when to use antibiotics and what indications they should be used for. Antibiotic knowledge scores are computed as the number of correct responses to 13 items, ranging between 0 and 13 as 1 was assigned to correct answers and zero for all other cases. Knowledge of antibiotic resistance assesses the level of understanding around the issue of antibiotic resistance, which was measured by computing the number of correct responses to 8 statements. The scores ranged between 0 and 8 as 1 was assigned to correct answers and zero for all other cases. The participants’ level of awareness of possible solutions to the problem of antibiotic resistance measures the level of the participants’ awareness of possible solutions to address the problem of antibiotic resistance. A 5-point Likert scale of 8 items was used to assess the participants’ agreement with a series of solutions to antibiotic resistance. Additionally, the participants’ perspectives on the scope of the antibiotic resistance problem measure the level of the participants’ awareness and understanding of how respondents believe the issue of antibiotic resistance is serious and whether it will have an impact on their health. A 5-point Likert scale of 6 items was used to assess the participants’ agreement with a series of statements connected to antibiotic resistance.

To verify face and content validity, the final survey was assessed by five professionals in clinical pharmacy and pharmacy practice. Because the survey was available in English, two native Arabic speakers translated it into Arabic to verify that an English equivalent version was obtained. Following back translation, two independent fluent English speakers, and native Arabic speakers, assessed the Arabic version’s optimal wording and structure. Twenty pilot study participants were recruited primarily to assess survey readability and comprehension, with the goal of standardizing the items provided to the participants. Following that, Cronbach’s alpha was calculated for the scales utilized in this study (see Table 7).

### 4.3. Statistical Analysis

The data collected in this study were analyzed using the IBM SPSS (Statistical Package for the Social Sciences) version 24.0 computer software. Descriptive analysis was used to report percentages, frequencies, means, and standard deviations of antibiotic knowledge and awareness scores obtained from the participants. In this study, a health literacy score of 1, 2, or 3 was classified as inadequate health literacy, whereas a score of 4 or 5 was classified as adequate health literacy. For subsequent analysis, knowledge of antibiotics was considered “inadequate” if the percentage scores were ≤50% (respondents’ score of 7 or less out of 13) and was considered “adequate” if the percentage scores >50%. Similarly, knowledge of antibiotic resistance was considered “inadequate” if the percentage scores were ≤50% (respondents’ score of 4 or less out of 8) and was considered “adequate” if the percentage scores >50%. The scores of both the participants’ perspectives on the scope of the antibiotic resistance problem and the level of awareness of possible solutions to the problem of antibiotic resistance were calculated by taking the average of the individuals’ responses to the multiple items on the scale as described by Kerlinger (1986) [62]; the scores ranged from 1 to 5. Since knowledge of antibiotic and antibiotic resistance was not following a normal distribution, a logistic regression model was then used to identify many factors (i.e., age, gender, residence, family structure, education level, employment status, family monthly income, health insurance, and infection with COVID-19) associated with adequate knowledge of antibiotics and antibiotic resistance. After completing univariate logistic regression, variables with *p* < 0.25 were included in the multiple logistic regression model (Supplementary Material Table S1) [63]. The adjusted odds ratios and 95% confidence intervals (CIs) were reported. The use of standard statistics (e.g., *t*-test and ANOVA) allowed this study to assess disparities across groups based on sociodemographic variables and to identify factors associated with the participants’ understanding of possible antibiotic resistance solutions and their perspectives on the scope of the antibiotic resistance problem. Following ANOVA testing, post hoc Tukey tests were performed to determine whether any of the groups varied substantially from the others. Data transformation was conducted using an inverse distribution function to ensure that data conformed to normality [64]. Levene’s statistics were used to test the assumption of variance homogeneity. The *p*-value was used to determine the statistical significance of all of the analyses, and significant findings were defined as a *p*-value of less than 0.05.

## Figures and Tables

**Table 1:** Percentage distribution of selected demographic variables of the participants in the sample (*n* = 1213).

Variable	*n* (%)
Age (years)	
18–24	279 (23)
25–34	429 (35.4)
35–44	334 (27.5)
>45	171 (14.1)
Gender	
Male	357 (29.4)
Female	856 (70.6)
Area of Living	
Urban	741 (61.1)
Suburban	242 (20)
Rural	230 (19)
Family Structure	
Nuclear family	607 (50)
Joint family	306 (25.2)
Extended family	300 (24.7)
Education Level	
Up to High School	256 (21.1)
Bachelor’s degree	666 (54.9)
Postgraduate degree (e.g., MSc, PhD)	291 (24)
Employment Status	
Unemployed	481 (39.7)
Employed in nonmedical field	389 (32.1)
Employed in medical field	343 (28.3)
Family Monthly Income	
Less than JOD 500 (USD 700)	399 (32.9)
JOD 501 (USD 700)–JOD 1000 (USD 1400)	549 (45.3)
JOD 1001 (USD 1400)–JOD 1500 (USD 2100)	147 (12.1)
Above JOD 1500 (USD 2100)	118 (9.7)
Do you have health insurance?	
Yes	931 (76.8)
No	282 (23.2)
Have you ever been infected with COVID-19?	
Yes	486 (40.1)
No	727 (59.9)

**Table 2:** Knowledge of antibiotic use among participants.

Statements	*n* (%)
When do you think you should stop taking antibiotics once you’ve begun treatment?	
When you feel better	208 (17.1)
When you’ve taken all of the antibiotics as directed	989 (81.5)
Don’t know	16 (1.3)
Do you think this statement is “true” or “false”? “It’s okay to use antibiotics that were given to a friend or family member, as long as they were used to treat the same illness.”	
True	165 (13.6)
False	997 (82.2)
Don’t know	51 (4.2)
Do you think this statement is “true” or “false”? “It’s okay to buy the same antibiotics, or request these from a doctor, if you’re sick and they helped you get better when you had the same symptoms before.”	
True	414 (34.1)
False	716 (59)
Don’t know	83 (6.8)
Do you think these conditions can be treated with antibiotics?	*n* of “Yes” Answers (%)
HIV/AIDS	57 (4.7)
Gonorrhea	173 (14.3)
Bladder infection or urinary tract infection	914 (75.4)
Diarrhea	202 (16.7)
Cold and flu	524 (43.2)
Fever	299 (24.6)
Malaria	158 (13)
Measles	85 (7)
Skin or wound infection	805 (66.4)
Sore throat	1021 (84.2)
Body aches	163 (13.4)
Headaches	169 (13.9)
COVID-19	319 (26.3)

**Table 3:** Knowledge of antibiotic resistance among participants.

Statement	Correct Answer	*n (%)*		
		False	I Don’t Know	True
Antibiotic resistance occurs when your body becomes resistant to antibiotics and they no longer work as well.	False	172 (14.2)	192 (15.8)	849 (70.0)
2. Many infections are becoming increasingly resistant to treatment by antibiotics.	True	149 (12.3)	222 (18.3)	842 (69.4)
3. If bacteria are resistant to antibiotics, it can be very difficult or impossible to treat the infections they cause.	True	363 (29.9)	299 (24.6)	550 (45.3)
4. Antibiotic resistance is an issue that could affect me or my family.	True	187 (15.4)	275 (22.7)	750 (61.8)
5. Antibiotic resistance is an issue in other countries but not in my country (Jordan).	False	568 (46.8)	394 (32.5)	250 (20.6)
6. Antibiotic resistance is only a problem for people who take antibiotics regularly.	False	519 (42.8)	281 (23.2)	412 (34.0)
7. Bacteria that are resistant to antibiotics can be spread from person to person.	True	361 (29.8)	402 (33.2)	449 (37.0)
8. Antibiotic-resistant infections could make medical procedures such as surgery, organ transplants, and cancer treatment much more dangerous.	True	76 (6.3)	304 (25.1)	832 (68.6)
Have you heard of any of the following terms before this survey?				*n* of “Yes” (%)
Antibiotic resistance				907 (74.8)
Superbugs				761 (62.7)
Antimicrobial resistance				583 (48.1)
Drug resistance				782 (64.5)
Antibiotic-resistant bacteria				830 (68.4)
Never heard of any of these terms				139 (11.5)

**Table 4:** Logistic regression of pattern of antibiotic usage associated with adequate knowledge of antibiotics and antibiotic resistance.

Statements	*n* (%)	Knowledge of Antibiotics (Adequate vs. Inadequate)		Knowledge of Antibiotic Resistance (Adequate vs. Inadequate)	
		OR (95% CI)	*p*-Value	OR (95% CI)	*p*-Value
When did you last take antibiotics?					
More than a year ago	188 (15.5)	3.63 (2.44–5.41)	0.000	2.08 (1.45–2.99)	0.000
Last year	119 (9.8)	2.19 (1.42–3.39)	0.000	2.13 (1.39–3.26)	0.001
Six months ago	365 (30.1)	1.74 (1.29–2.35)	0.000	1.42 (1.06–1.91)	0.02
One month ago	344 (28.4)	1		1	
On that occasion, did you get the antibiotics by a prescription from a doctor or nurse? (Appropriate self-medication of antibiotics)					
Yes	851 (70.2)	2.01 (1.57–2.58)	0.000	1.31 (1.03–1.68)	0.03
No or can’t remember	362 (29.8)	1		1	
On that occasion, did you get advice from a doctor, nurse, or pharmacist on how to take them?					
Yes	889 (73.3)	1.98 (1.53–2.56)	0.000	1.18 (0.91–1.52)	0.215
No or can’t remember	324 (26.7)	1		1	
Main sources of self-medication antibiotics (*n* = 362)					
Pharmacy	185 (51.1)				
Healthcare centers	45 (12.4)				
Friends or family	88 (24.3)				
Using leftover antibiotics	44 (12.2)				

**Table 5:** Multiple logistic regression of sociodemographic factors and levels of health literacy associated with participants’ knowledge of antibiotics and antibiotic resistance.

Variables	Knowledge of Antibiotics (Adequate vs. Inadequate)			Knowledge of Antibiotic Resistance (Adequate vs. Inadequate)		
	Standardized β (S.E.)	Adjusted OR (95% CI)	*p*-Value *	Standardized β (S.E.)	Adjusted OR (95% CI)	*p*-Value *
Age						
18–24	0.9 (0.23)	2.46 (1.55–3.9)	0.000	0.29 (0.22)	1.34 (0.87–2.08)	0.179
25–34	0.48 (0.22)	1.61 (1.05–2.49)	0.029	0.29 (0.2)	1.34 (0.9–1.99)	0.148
35–44	0.18 (0.22)	1.2 (0.77–1.86)	0.401	0.38 (0.2)	1.46 (0.98–2.18)	0.062
>45	Reference			Reference		
Gender						
Male	−0.39 (0.14)	0.67 (0.5-0.89)	0.007	−0.11 (0.17)	0.89 (0.69–1.14)	0.364
Female (Ref.)		Reference			Reference	
Area of Living						
Urban	0.4 (0.17)	1.5 (1.07–2.10)	0.018	0.98 (0.67–1.41)	0.928	0.269
Suburban	−0.04 (0.2)	0.96 (0.64–1.43)	0.847	1.15 (0.85–01.54)	0.361	
Rural	Reference			Reference		
Employment Status						
Employed in medical field	1.12 (0.18)	3.08 (2.13–4.44)	0.000	1.02 (0.16)	2.78 (2.00–3.87)	0.000
Employed in nonmedical field	−0.03 (0.16)	0.96 (0.69–1.33)	0.822	−0.03 (0.15)	0.97 (0.71–1.30)	0.849
Unemployed	Reference			Reference		
Do you have health insurance?						
Yes	0.15 (0.15)	1.16 (0.85–1.57)	0.332	0.32 (0.14)	1.38 (1.03–1.85)	0.026
No (Ref.)		Reference			Reference	
How do you protect yourself and others from COVID-19?						
Received antibiotics?						
Yes (n = 250, 20.6%)	−1.11 (0.18)	0.31 (0.22–0.45)	0.000	−0.29 (0.17)	0.74 (0.53–1.05)	0.093
No	Reference			Reference		
Received COVID-19 vaccine?						
Yes (n = 377, 31.1%)	0.48 (0.15)	1.62 (1.20–2.18)	0.002	0.22 (0.14)	1.24 (10.94–1.64)	0.130
No	Reference			Reference		
Health Literacy						
Adequate (n = 759, 62.6%)	0.31 (0.13)	1.37 (1.05–1.77)	0.017	0.38 (0.12)	1.46 (1.14–1.89)	0.003
Inadequate	Reference			Reference		

* Significance at *p* < 0.05; one-way ANOVA or *t*-test; SD: standard deviation.

**Table 6:** Participants’ awareness and perspectives concerning antibiotics in relation to variables of interest.

Variables	Awareness of Possible Solutions to the Problem of Antibiotic Resistance		Perspectives on the Scope of the Antibiotic Resistance Problem	
	Mean (SD)	*p*-Value *	Mean (SD)	*p*-Value *
Age				
18–24	3.89 (0.97)	0.004	3.42 (0.76)	0.066
25–34	3.99 (0.89)		3.51 (0.70)	
35–44	3.88 (0.95)		3.57 (0.70)	
>45	3.68 (0.94)		3.49 (0.71)	
Gender				
Male	3.85 (0.99)	0.271	3.45 (0.72)	0.071
Female	3.92 (0.91)		3.53 (0.72)	
Area of Living				
Urban	3.95 (0.93)	0.061	3.53 (0.71)	0.181
Suburban	3.81 (0.95)		3.46 (0.72)	
Rural	3.82 (0.92)		3.45 (0.73)	
Family Structure				
Nuclear family	3.89 (0.95)	0.846	3.54 (0.76)	0.162
Joint family	3.92 (0.95)		3.48 (0.71)	
Extended family	3.89 (0.9)		3.45 (0.63)	
Education Level				
Up to high school	3.77 (0.92)	0.002	3.49 (0.77)	0.489
Bachelor’s degree	3.88 (0.94)		3.52 (0.73)	
Postgraduate degree	4.05 (0.93)		3.46 (0.65)	
Employment Status				
Employed in medical field	3.92 (0.96)	0.156	3.57 (0.74)	0.828
Employed in nonmedical field	3.89 (0.95)		3.47 (0.7)	
Unemployed	3.88 (0.9)		3.48 (0.72)	
Family Monthly Income				
Less than JOD 500	3.89 (0.93)	0.742	3.47 (0.72)	0.650
JOD 501–JOD 1000	3.87 (0.96)		3.52 (0.73)	
JOD 1001–JOD 1500	3.96 (0.96)		3.54 (0.73)	
Above JOD 1500	3.94 (0.81)		3.5 (0.65)	
Do you have health insurance?				
Yes	3.89 (0.94)	0.822	3.52 (0.72)	0.157
No	3.91 (0.92)		3.45 (0.71)	
Have you ever been infected with COVID-19?				
Yes	3.91 (0.91)	0.614	3.50 (0.69)	0.896
No	3.89 (0.95)		3.51 (0.74)	
Health Literacy				
Adequate	3.95 (0.93)	0.011	3.54 (0.72)	0.016
Inadequate	3.81 (0.94)		3.44 (0.71)	
Knowledge of Antibiotics				
Adequate	3.93 (0.94)	0.003	3.53 (0.71)	0.001
Inadequate	3.71 (0.91)		3.34 (0.73)	
Knowledge of Antibiotic Resistance				
Adequate	4.01 (0.99)	0.000	3.63 (0.74)	0.000
Inadequate	3.78 (0.86)		3.36 (0.67)	

* Significance at *p* < 0.05; one-way ANOVA or *t*-test; SD: standard deviation.

**Table 7:** Internal consistency of main concepts.

Subscales	No. of Items	Mean ± SD	Cronbach’s α
Knowledge of antibiotics	15	9.76 ± 2.06	0.67
Knowledge of antibiotic resistance	8	4.42 ± 2.03	0.65
Awareness of possible solutions to the problem of antibiotic resistance	8	3.90 ± 0.97	0.94
Perspectives on the scope of the antibiotic resistance problem	6	3.51 ± 0.73	0.82

## Data Availability

Data are available on reasonable request and in line with permission approval processes from the Jordan University of Science and Technology.

## Supplementary Materials

The following are available online at https://www.mdpi.com/article/10.3390/antibiotics10091107/s1: Table S1: Simple logistic regression of sociodemographic factors and levels of health literacy associated with participants’ knowledge of antibiotics and antibiotic resistance.
