# Supplementary material for: Public Health Literacy, Knowledge, and Awareness Regarding Antibiotic Use and Antimicrobial Resistance during the COVID-19 Pandemic: A Cross-Sectional Study
Source: Antibiotics (Basel). 2021 Sep 13;10(9):1107. doi: 10.3390/antibiotics10091107 (PMC8472776; doi:10.3390/antibiotics10091107)
Supplement: Supplementary file 1 [file antibiotics-10-01107-s001.zip › antibiotics-1335070-supplementary.pdf]

antibiotics

MDPI

Table S1. Simple logistic regression of sociodemographic factors and levels of health literacy associated with participants' knowledge of antibiotics and antibiotic resistance.

	**Knowledge of Antibiotics** (Adequate VS Inadequate)		**Knowledge of Antibiotic Resistance** (Adequate VS Inadequate)	
	OR (95% CI)	*P*-Value	OR (95% CI)	*P*-Value
**Age**				
18-24	2.94 (1.96-4.34)	0.000	1.02(0.69-1.49)	0.913
25-34	1.56 (1.07-2.27)	0.024	1.34(0.93-1.91)	0.107
35-44	1.15 (0.77-1.69)	0.521	1.44(0.99-2.09)	0.051
> 45 (Ref.)	1		1	
**Gender**				
Male	0.79 (0.61-1.01)	0.069	0.98(0.77-1.26)	0.929
Female (Ref.)	1		1	
**Area of Living**				
Urban	1.63 (1.21-2.2)	0.001	1.14(0.85-1.54)	0.361
Suburban	0.99 (0.69-1.42)	0.981	0.98(0.68-1.41)	0.928
Rural	1		1	
**Family Structure**				
Nuclear family	0.93 (0.7-1.24)	0.657	1.17(0.89-1.55)	0.252
Joint family	0.83 (0.6-1.14)	0.263	0.94(0.69-1.30)	0.746
Extended family (Ref.)	1		1	
**Educational Level**				
Postgraduate degree	1.42 (1.01-2)	0.042	2.04 (1.45-2.88)	0.000
Bachelor's degree	1.25 (0.94-1.68)	0.122	1.64(1.22-2.19)	0.001
Up to High School (Ref.)	1		1	
**Employment Status**				
Employed in medical field	3.88 (2.82-5.33)	0.000	3.79 (2.8-5.12)	0.000
Employed in non-medical field	1.13 (0.86-1.48)	0.362	1.13(0.86-1.48)	0.369
Unemployed (Ref.)	1		1	
**Total Family Income**				
Above JOD 1,500	1.89 (1.22-2.93)	0.004	2.06(1.35-3.14)	0.001
JOD 1,001-JOD 1,500	1.61(1.08-2.38)	0.018	2.02(1.37-2.97)	0.000
JOD 501-JOD 1,000	1.19 (0.92-1.55)	0.179	1.56(1.2-2.03)	0.001
Less than JOD 500 (Ref.)	1		1	
**Do You Have Health Insurance**				
Yes	1.44 (1.1-1.89)	0.007	1.77(1.35-2.32)	0.000
No (Ref.)	1		1	
**Have you ever infected with COVID-19**				
Yes	1.03 (0.81-1.3)	0.81	1.77(1.35-2.32)	0.000
No (Ref.)	1		1	
**Protect yourself and others from COVID-19**				
Received Antibiotics? Yes (n=250, 20.6%)	0.32 (0.227-0.45)	0.000	0.65(0.47-0.91)	0.009
No (Ref.)	1		1	
Social distancing and wearing facemask?	0.84 (0.66-1.06)	0.154	0.94(0.61-1.45)	0.779

Yes (n=490, 40.4%)				
No (Ref.)	1		1	
Registered my name to receive COVID-19 vaccine?				
Yes (n= 463, 38.2%)	0.78 (0.62-0.99)	0.039	0.98(0.78-1.25)	0.92
No (Ref.)	1		1	
Received COVID-19 Vaccine?				
Yes (n=377, 31.1%)	2.18 (1.7-2.86)	0.000	1.54(1.21-1.97)	0.001
No (Ref.)	1		1	
**Health Literacy**				
Adequate (n= 759, 62.6%)	1.73 (1.36-2.19)	0.003	1.79(1.41-2.26)	0.000
Inadequate (Ref.)	1		1	
